# Cerebral and systemic physiological effects of wearing face masks in young adults

**DOI:** 10.1073/pnas.2109111118

**Published:** 2021-10-04

**Authors:** Jonas B. Fischer, Lisa Kobayashi Frisk, Felix Scholkmann, Raquel Delgado-Mederos, Mercedes Mayos, Turgut Durduran

**Affiliations:** ^a^ICFO - Institut de Ciències Fotòniques, Barcelona Institute of Science and Technology, Castelldefels (Barcelona) 08860, Spain;; ^b^Biomedical Optics Research Laboratory, Department of Neonatology, University Hospital Zurich, University of Zurich, Zurich 8091, Switzerland;; ^c^Sant Pau Biomedical Research Institute, Department of Neurology, Hospital de la Santa Creu i Sant Pau, Barcelona 08041, Spain;; ^d^Sleep Unit, Department of Respiratory Medicine, Hospital de la Santa Creu i Sant Pau, Barcelona 08041, Spain;; ^e^Centro de Investigación Biomédica en Red (CIBER) Enfermedades Respiratorias (CB06/06), Madrid 28029, Spain;; ^f^Institució Catalana de Recerca i Estudis Avançats, Barcelona 08010, Spain

**Keywords:** face masks, cerebral hemodynamics, systemic physiology, COVID-19, near-infrared spectroscopy

## Abstract

The COVID-19 pandemic led to widespread mandates requiring the wearing of face masks, which led to debates on their benefits and possible adverse effects. To that end, the physiological effects at the systemic and at the brain level are of interest. We have investigated the effect of commonly available face masks (FFP2 and surgical) on cerebral hemodynamics and oxygenation, particularly microvascular cerebral blood flow (CBF) and blood/tissue oxygen saturation (StO_2_), measured by transcranial hybrid near-infrared spectroscopies and on systemic physiology in 13 healthy adults (ages: 23 to 33 y). The results indicate small but significant changes in cerebral hemodynamics while wearing a mask. However, these changes are comparable to those of daily life activities. This platform and the protocol provides the basis for large or targeted studies of the effects of mask wearing in different populations and while performing critical tasks.

Many governments have mandated the wearing of face masks in response to the coronavirus disease 2019 (COVID-19) pandemic in order to mitigate the acute respiratory syndrome coronavirus 2 (SARS-CoV-2) transmission. The effectiveness of this measure is currently being evaluated (1). This has led to ongoing discussions about possible adverse effects of mask wearing (e.g., dizziness, headaches, fainting), especially within the elderly, during long-term continuous mask usage and during physical activity. Chan et al. (2) reported that the arterial oxygenation (SpO_2_) did not change in elderly subjects after 1 h, while Law et al. (3) reported a significant effect on baseline cerebral hemodynamics and end-tidal carbon dioxide pressure (EtCO_2_) using functional MRI (fMRI) on middle-aged adults. No task-induced hemodynamic changes were found in this study. The bulk of these concerns arise due to potential hypercapnic effects of carbon dioxide rebreathing, which has not yet been evaluated in a thorough manner. Also, the brain function was evaluated only at the level of a surrogate of oxygen consumption. Noninvasive functional near-infrared spectroscopy (fNIRS) and functional diffuse correlation spectroscopy (fDCS) use near-infrared light to measure microvascular cerebral hemodynamics without the constraints of the fMRI scanners. When combined together, they allow us to relate cerebral blood/tissue oxygen saturation and blood flow to the cerebral oxygen metabolism. Their main disadvantage is the potential signal contamination due to the extracerebral tissues and the limited penetration depth. Nevertheless, the advantage of studying mask effects on brain function in realistic settings merits their uses for a thorough study to look at the physiology in a holistic manner.

To this end, we have investigated the effect of mask wearing (FFP2 [European Union standard, similar to N95 in North America and KN95 in China] versus surgical) on cerebral hemodynamics, blood/tissue oxygenation, and oxygen metabolism as well as the systemic physiology with a multimodal platform of custom near-infrared spectroscopies and commercial physiological monitors in healthy young adults.

## Results

**Fig. 1.**
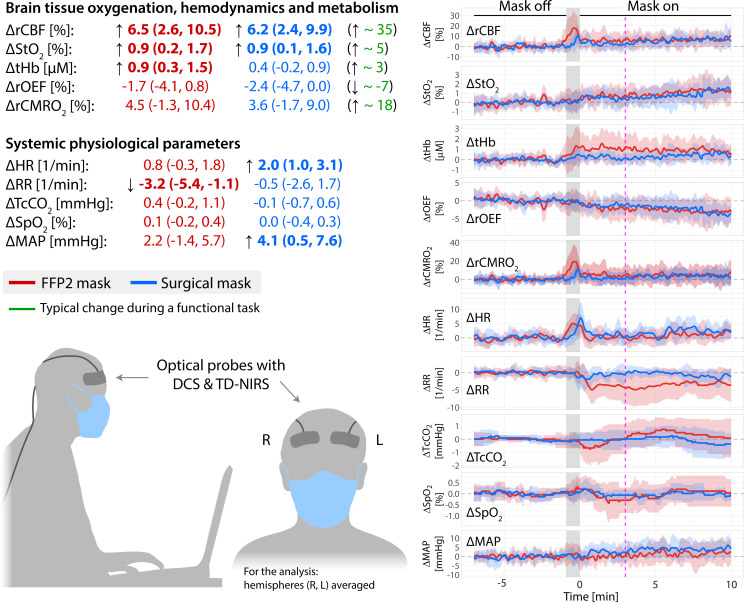
(*Left*) Changes in the different parameters are shown for FFP2 masks (red) and surgical masks (blue). “Δ” denotes a difference and “*r*” is a ratio. The arrow indicates a significant change (*P* < 0.05) whose direction is an increase or a decrease. Additionally, for cerebral hemodynamics, we have reported (green) changes during typical tasks such as basic cognitive, visual, or motor tasks (4, 8) for comparison. (*Right*) Time series of the population mean of all parameters are shown for both mask types (same color code). Time 0 is the time when the mask was placed and the shaded area indicates the time taken for placing the mask, which was excluded from the analysis. Data to the *Right* of the 3-min mark (magenta) were used for analysis to allow the physiology to stabilize after placing the mask. The data were normalized to 300 s prior to the mask placement.

Thirteen volunteers (median age: 27.0 y [23 to 33 y], six females) took part in the study. Fig. 1 summarizes the findings. Small but significant changes in cerebral blood flow (CBF) and cerebral blood oxygen saturation (StO_2_) were detected for both mask types: 1) CBF increased by 6.5% (95% CI: 2.6, 10.5%) for the FFP2 mask and 6.2% (95% CI: 2.4, 9.9%) for the surgical mask; 2) StO_2_ increased by 0.9% (95% CI: 0.2, 1.7%) for the FFP2 mask and also 0.9% (95% CI: 0.1, 1.6%) for the surgical mask; 3) total hemoglobin concentration (tHb) increased significantly only for the FFP2 mask by 0.9 µM (95% CI: 0.3, 1.5 µM). Changes in oxygen extraction fraction (OEF) and cerebral metabolism (CMRO_2_) (defined in *SI Appendix*) were not statistically significant: 1) OEF decreased by −1.7% (85% CI: −4.1, 0.8) for the FFP2 mask and by −2.4% (95% CI: −4.7, 0.0) for the surgical mask; 2) CMRO_2_ increased by 4.5% (95% CI: −1.3, 10.4%) for the FFP2 mask and by 3.6% (95% CI: −1.7, 9.0%) for the surgical mask. None of these changes were statistically significantly different between the two mask types.

EtCO_2_ showed a significant change but was discarded since the probe was affected by the air trapped within the mask. Transcutaneous carbon dioxide partial pressure (TcCO_2_) and SpO_2_ did not significantly change due to wearing a mask, while mean arterial pressure (MAP) and heart rate (HR) increased significantly for the surgical mask by 4.1 mmHg (95% CI: 0.5, 7.6 mmHg) and 2.0 beats/min (95% CI: 1.0, 3.1 beats/min), respectively. Respiratory rate (RR) decreased significantly for the FFP2 mask by 3.2 breaths/min (95% CI: −5.4, −1.1 breaths/min). A significant difference in HR between mask types of 1.2 beats/min (95% CI: 0.0, 2.4 beats/min) was detected.

## Discussion

Our findings show that wearing a face mask leads to statistically significant changes in the cerebral hemodynamics and oxygenation (CBF and StO_2_) in healthy young subjects at rest, even for this first relatively short period of mask usage. However, the changes observed are minimal and are comparable to those typically observed during daily life (4). Within the limitations of the study, we cannot claim any concerns for mask use during daily life activities for healthy, young individuals. In order to draw a stronger conclusion, the duration of mask wearing could have been longer (harder to disentangle its effects from other physiological variables such as fatigue), the study population should be more heterogeneous representing the society in general, and the sample sizes can be increased. Another limitation is the fact that the order of the masks was fixed, therefore one should be critical about the results regarding differences in the mask types and additional differences may be revealed in the future. The noticeable differences in variance of the time traces are related to the intersubject variability, which may be related to the fit of the mask, mask types, and the individual’s physiology.

Furthermore, we did not observe significant changes in TcCO_2_ and SpO_2_. The increase in MAP and HR for the surgical mask may have been caused by the discomfort of probes, placement of masks, and the order of studies. Here, we did not account for these stressors as potential confounding effects, since they are also part of daily life. This observation is further strengthened since TcCO_2_ did not change, i.e., the hypothesized hypercapnic effect was not observed. We stress that in the literature mainly EtCO_2_ is utilized as a surrogate of blood carbon dioxide levels, which, however, is influenced by the trapped carbon dioxide under the mask with standard equipment (5). TcCO_2_ provides insights as a better surrogate for the partial pressure of carbon dioxide.

Overall, our study provides a holistic view of understanding the potential effects of mask wearing in healthy, young adults by a thorough characterization of both the systemic physiology, the presumed driving biomarker of carbon dioxide rebreathing effect, and cerebral hemodynamics. The large intersubject variability while wearing a mask suggests that individuals may have differing responses and the platform/protocol that we introduce here could be utilized on elderly subjects or those with preexisting respiratory or cerebrovascular problems. These populations may behave differently. Finally, the potential effect of mask wearing on individuals performing critical tasks needs to be studied with future investigations. Investigations of these effects are important for policy making in order to maintain quality of life for individuals and for minimizing risks in persons carrying out critical tasks.

## Materials and Methods

The study protocol was approved by the ethical committee of Hospital Clinic Barcelona and all participants signed informed consent. Young healthy adults (range for inclusion: 20 to 35 y of age) were recruited. Participants sat in a chair and read a scientific text during the experiments. The experimental paradigm involved two 10-min periods: 1) without wearing a mask and 2) with a mask. A commonly used three-layer surgical mask and a FFP2 mask (RM101 FFP2 NR, Zhejiang Yinghua Technology Co. Ltd.) were tested. Cerebral blood flow, oxygenation, and oxygen metabolism were measured bilaterally over the prefrontal cortex using transcranial diffuse correlation spectroscopy (DCS) and time-resolved near-infrared spectroscopy (TR-NIRS) (6). Changes in CBF, StO_2_, and tHb were determined. MAP, HR, SpO_2_, RR, EtCO_2_, and TcCO_2_ were monitored. Signal processing was performed with Matlab (R2019a, MathWorks) and statistical analysis (R, v4.0.3) was applied to determine whether mask wearing was leading to a significant change in the signals. Raw DCS and TR-NIRS data were fitted using the analytical solution. Artifacts were manually removed, the data were smoothed (30-s window), and the changes were averaged over both hemispheres since no difference between them was detected (*P* >> 0.5, paired Wilcoxon test). For further details see *SI Appendix*.

## Supplementary Material

Supplementary File

## Data Availability

Data for analysis and plots as txt files R code for analysis and plot have been deposited in Zenodo (10.5281/zenodo.4775432) (https://doi.org/10.5281/zenodo.4775432) (7).
