# Supplementary material for: Cerebral and systemic physiological effects of wearing face masks in young adults
Source: Proc Natl Acad Sci U S A. 2021 Oct 4;118(41):e2109111118. doi: 10.1073/pnas.2109111118 (PMC8522266; doi:10.1073/pnas.2109111118)
Supplement: Supplementary File [file pnas.2109111118.sapp.pdf]

Supplementary Information for

Cerebral and Systemic Physiological Effects of Wearing Face Masks in Young Adults

Jonas B. Fischer[1,†]; Lisa Kobayashi Frisk[1, †]; Felix Scholkmann[2]; Raquel Delgado-Mederos[3]; Mercedes Mayos[4,5]; Turgut Durduran[1,6,*]

[1] ICFO-Institut de Ciències Fotòniques, The Barcelona Institute of Science and Technology, Castelldefels (Barcelona), Spain
[2] University Hospital Zurich, University of Zurich, Department of Neonatology, Biomedical Optics Research Laboratory, Zurich, Switzerland
[3] Hospital de la Santa Creu i Sant Pau, Department of Neurology, Sant Pau Biomedical Research Institute, Barcelona, Spain
[4] Hospital de la Santa Creu i Sant Pau, Department of Respiratory Medicine, Sleep Unit, Barcelona, Spain
[5] CIBER Enfermedades Respiratorias (CibeRes) (CB06/06), Madrid, Spain
[6] Institució Catalana de Recerca i Estudis Avançats (ICREA), Barcelona, Spain
† Co-first authors with equal contribution.
* Corresponding author

This file includes:

SI Extended Methods

Supplementary Information/ Extended Methods

Participants were recruited from the general population of a research institute adhering to COVID-19 regulations. Therefore, we have aimed to recruit healthy young volunteers in an age range between 20 and 35 years. We have ruled out existing cardiovascular, neurological or respiratory conditions as assessed by a questionnaire. All subjects were measured with two different masks on two separate days – on day one an FFP2 mask (RM101 FFP2 NR, Zhejiang Yinghua Technology Co. Ltd., China) was utilized and a three-layer surgical mask was utilized on day two. The order of the masks was fixed.

Diffuse optical technologies using near-infrared (~650-950nm) are emerging non-invasive methods that can measure microvascular blood flow (CBF), blood oxygen saturation (StO_2) and total hemoglobin concentration (tHb) continuously on the surface of the adult brain cortex [1]. Here, we have used diffuse correlation spectroscopy (DCS) [2] and time-resolved near-infrared spectroscopy (TR-NIRS) [3] to measure these parameters simultaneously and bilaterally with a source-detector separation of 2.5 cm for DCS and 3.0 cm for TR-NIRS. The probes were placed on two frontal lobes while avoiding the sinuses.

Alongside the cerebral hemodynamic assessment, systemic physiology was monitored in a synchronized manner using both hardware and software protocols. Arterial blood pressure (ABP), heart rate (HR), arterial oxygen saturation (SpO_2) were monitored with a non-invasive finger cuff (Finapres NOVA, Finapres Medical Systems BV, Netherlands). Respiratory rate (RR) and exhaled end-tidal carbon dioxide partial pressure ($EtCO_2$) by a capnograph (Capnostream 20p, Medtronic, Minneapolis, USA), and, transcutaneous carbon dioxide partial pressure ($TcCO_2$) by a transcutaneous monitor (SenTec Digital monitor, SenTec AG, Switzerland) were monitored. During the study, we have expected and confirmed that the nasal cannula to measure $EtCO_2$ is influenced by the carbon dioxide trapped between face and mask and its readings do not reflect the blood carbon-dioxide levels so $TcCO_2$ measurements were utilized.

The optical measurement of microvascular CBF, microvascular StO_2 and SpO_2 allows us to further calculate changes in the oxygen extraction fraction (OEF) (see equation 1) and the cerebral metabolic rate of oxygen ($CMRO_2$) (see equation 2). This calculation is based on a set of assumptions that are derived from a compartmental model where the vasculature is divided into arterial, capillary and venous compartments [1,4] and has been compared against various methods [5], resulting in:

$$OEF = \frac{SaO_2 - StO_2}{\gamma \cdot SaO_2} \quad (1) \text{ and}$$

$$rCMRO_2 = rOEF \cdot rCBF \sim \frac{SaO_{2,0} \cdot (SaO_2 - StO_2)}{SaO_2 \cdot \left(SaO_{2,0} - StO_{2,0}\right)} \cdot \frac{CBF}{CBF_0}. \quad (2)$$

Here γ is the fraction of the blood volume in the venous compartment, which is assumed not to change, r denotes relative changes with respect to the baseline indicated with index 0 and SaO_2 is the arteriolar oxygen saturation which can be estimated by SpO_2. The limitations and applicability of these assumptions have been discussed in the references and are beyond the scope of this study.

The calculation of the optically measured variables and the re-alignment with the other physiological signals was done in MATLAB (R2019a, MathWorks, USA). DCS and TR-NIRS data was fitted using the analytical solutions for a semi-infinite medium [2,3]. All parameters were smoothed with the a robust, “local regression using weighted linear least squares and a second degree polynomial model“ (“`rloess`” method of the function “`smoothdata`”) using a thirty second window to reduce physiological noise [6]. The changes in the cerebral hemodynamic parameters of both hemispheres were then averaged since the results from the two hemispheres were not statistically significantly different ($p \gg 0.05$, paired Wilcoxon test between hemispheres). The signals were visually inspected and artifacts (spikes, jumps, etc.) were manually removed (periods in time) [7].

The statistical analysis was performed using the statistical programming language R (v 4.0.3) with the packages “`lmer`” and “`emmeans`”. In order to test for changes in the hemodynamic and systemic parameters and differences between the two mask types a linear mixed effect (LME) model was used with the subject as the random effect. We have analyzed the data after three minutes of

wearing a mask to allow the physiology to stabilize and compared it to a baseline 300 seconds prior to the mask placement. We report the results of a post-hoc test. We have tested whether each mask type is statistically different from zero and whether the difference between the mask types is statistically different considering the “false discovery rate” correction for the p-value (<0.05 significant) due to multiple testing. The residuals of the LME were visually inspected if they can be considered to be normally distributed.

SI References

1. Durduran T, Choe R, Baker WB, Yodh AG. Diffuse optics for tissue monitoring and tomography. Reports on Progress in Physics. 2010;73(7):076701.
2. Durduran T, Yodh AG. Diffuse correlation spectroscopy for non-invasive, micro-vascular cerebral blood flow measurement. Neuroimage. 2014;85:51-63.
3. Torricelli A, Contini D, Pifferi A, Caffini M, Re R, Zucchelli L, Spinelli L. Time domain functional NIRS imaging for human brain mapping. Neuroimage. 2014;85:28-50.
4. Culver JP, Durduran T, Furuya D, Cheung C, Greenberg JH, Yodh AG. Diffuse optical tomography of cerebral blood flow, oxygenation, and metabolism in rat during focal ischemia. Journal of cerebral blood flow & metabolism. 2003;23(8):911-24
5. Jain V, Buckley EM, Licht DJ, Lynch JM, Schwab PJ, Naim MY, Lavin NA, Nicolson SC, Montenegro LM, Yodh AG, Wehrli FW. Cerebral oxygen metabolism in neonates with congenital heart disease quantified by MRI and optics. Journal of Cerebral Blood Flow & Metabolism. 2014;34(3):380-8.
6. Jahani S, Setarehdan SK, Boas DA, Yücel MA. Motion artifact detection and correction in functional near-infrared spectroscopy: a new hybrid method based on spline interpolation method and Savitzky–Golay filtering. Neurophotonics. 2018;5(1):015003.
7. Selb J, Wu KC, Sutin J, Farzam P, Bechek S, Shenoy A, Patel AB, Boas DA, Franceschini MA, Rosenthal ES. Prolonged monitoring of cerebral blood flow and autoregulation with diffuse correlation spectroscopy in neurocritical care patients. Neurophotonics. 2018;5(4):045005.
